# Physical characterization of late-type contact binary systems observed by LAMOST: a comprehensive statistical analysis

**DOI:** 10.1038/s41598-023-48507-5

**Published:** 2023-12-08

**Authors:** H. I. Abdel Rahman, Mohamed Darwish

**Affiliations:** Astronomy Department, National Research Institute of Astronomy and Geophysics, Helwan, Cairo 11421 Egypt

**Keywords:** Astronomy and astrophysics, Astronomy and planetary science

## Abstract

This paper presents a catalog of approximately 1800 Eclipsing W UMa systems (EWs) using parameters from LAMOST, VSX, ZTF and Gaia. Our detailed statistical analysis includes frequency distributions of parameters, confidence intervals, and hypothesis testing to provide deeper insights into the physical properties of this important eclipsing binary class. We focus on key parameters, including Period, Effective Temperature, Surface Gravity, metallicity, Radial Velocity, and spectral type of the systems. Our study reveals that the mean values for period, effective temperature, logarithmic surface gravity, metallicity, and radial velocity for EW systems are 0.377 days, 5775 K, 4, -0.185, and -4.085 km/s, respectively. The 95% confidence intervals for these parameters are 0.372 to 0.382 days, 5730 to 5820 K, -0.202 to -0.168, 3.97 to 4.03, and -6.47 to -1.7 km/s, respectively. Hypothesis testing of the estimated intervals results in the acceptance of the null hypothesis, indicating that EW systems are characterized within the specified limits. Our study also confirms that the majority of EW systems are late-type stars, primarily classified as F spectral type, followed by G and K. Interestingly, among the sample, 88 systems are classified as A spectral type, with a mean surface temperature of 7400 K. We examine the correlation between orbital periods and atmospheric parameters in the VSX and ZTF catalogs. While ZTF periods align well with established relations (correlation coefficient: 0.74), a weaker correlation is found in the VSX catalog. This highlights the need for a revision of VSX periods for improved accuracy in the studied sample of EWs.

## Introduction

Within the vast celestial canvas, binary star systems serve as captivating enigmas, offering crucial insights into stellar evolution and fundamental astrophysical processes. Among these intriguing binary configurations, the Late-Type Contact Binary systems (CBs), specifically belonging to the W Ursae Majoris (W UMa) class, hold a special significance. W UMa variables, constitute a fascinating class of binary star systems where two late-type dwarfs come into intimate contact, sharing a common convective envelope that lies between their inner and outer critical Roche-lobe surfaces. With orbital periods shorter than one day, these variables exhibit continuous light variation, making it challenging to precisely determine the onset and end of eclipses. Remarkably, the depths of the primary and secondary minima are nearly equal, implying that both components possess nearly identical temperatures and are in thermal contact, distinguishing them from EB-type binaries (e.g.^1,2^). While EW-type binaries are frequently detected in older open clusters and globular clusters, they are conspicuously absent in young stellar clusters^3^.

Despite extensive research from both photometric (e.g.^4–10^) and spectroscopic (e.g.^11–14^) modes, the formation mechanism of EW binaries remains enigmatic. It is postulated that they may evolve from short-period detached binaries through angular momentum loss via magnetic braking over timescales of a few hundred million to a few billion years (^15,16^). Additionally, the involvement of third bodies in the early dynamical interaction and later evolution of these systems may play a crucial role in their origin (e.g.^17,18^).

Late-type contact binary systems observed by the Large Sky Area Multi-Object Fiber Spectroscopic Telescope (LAMOST) have emerged as an intriguing subset of W UMa variables, offering valuable insights into stellar evolution and fundamental astrophysical processes. Due to their close proximity, mass and energy transfer between the components play a crucial role in shaping their properties and behavior. To gain a comprehensive understanding, a rigorous statistical analysis of a significant sample size is essential. Previous studies have explored various characteristics of CBs, including their common envelope and similar component temperatures (e.g.^19–22^). However, the presence of periodic thermal-relaxation oscillations in some systems adds complexity to their evolutionary paths (e.g.^23^). Moreover, a key puzzle remains unresolved—the existence of an evolutionary sequence among different types of CBs, which calls for larger sample sizes to establish conclusive trends.

A more extensive dataset is indispensable to refine evolutionary models, examine angular-momentum loss properties, and unveil the nuclear evolutionary pathways that impact orbital periods and the resulting evolutionary products of distinct CB types. Recent strides in observational capabilities, facilitated by sky surveys such as SuperWASP, ASAS-SN, NSVS, ZTF, GAIA, LAMOST and ATLAS (^24–29^), have substantially expanded the known sample of CBs . These invaluable data resources have empowered researchers to construct genuine and comprehensive CB samples, setting the stage for further statistical analyses and investigations into these captivating binary star systems.

In this paper, we undertake a comprehensive statistical analysis of approximately 1800 W Ursae Majoris (W UMa) systems gathered from the Large Sky Area Multi-Object Fiber Spectroscopic Telescope Data Release 7 (LAMOST DR7). Our investigation focuses on key parameters such as Period, effective temperature, surface gravity, metallicity, and radial velocity, this parameters also known as atmospheric parameters. By delving into these crucial aspects, we aim to gain deeper insights into the evolutionary behavior and asymmetry exhibited by the EW UMa systems. The findings from this study are expected to significantly contribute to our understanding of these fascinating binary star systems. Section “Data” provides an overview of the source of our sample data. In Section “Statistical analysis”, we elaborate on the statistical method employed to derive our findings. Lastly, Section “Discussion and conclusions” presents our discussion and conclusions based on the study’s outcomes.

## Data

We gathered our sample data from the LAMOST DR7 V2.0 [http://dr7.lamost.org/] catalogue and conducted a cross-match with the VSX (Variable Star Index), ZTF (Zwicky Transient Facility) variable star catalog (^30^) and GAIA DR3 (Global Astrometric Interferometer for Astrophysics Data Release3) to determine the period, system IDs and distance (in Kpc) of the stars in our study. The criterion for identification involved ensuring that this offset was less than 2 arcseconds. For this investigation, we specifically selected the LAMOST LRS Stellar Parameter Catalog of A, F, G, and K Stars, which is expected to encompass the EW systems of interest. The LAMOST, also known as the Guoshoujing Telescope, is a remarkable 4-meter quasi-meridian reflecting Schmidt telescope equipped with 4000 fibers, allowing simultaneous spectroscopic observations within its expansive 5° field of view. Notably, starting in 2017, new medium-resolution spectrographs with a resolving power of R = 7500 were incorporated alongside the existing low-resolution spectrographs (R = 1800)^31^. Atmospheric parameters and spectral classes are determined for the observed objects automatically by LASP (LAMOST stellar parameter pipeline)^32^. This automated process relies on the Universite de Lyon spectroscopic analysis software (ULySS) developed by^33^. Utilizing empirical spectral libraries, such as ELODIE, and an implemented *interpolator* function called TGM (^34,35^), ULySS accurately fits the whole observed spectra. According to^32^, the intrinsic external accuracies derived for high-quality AFGK stellar spectra using ULySS are 43 K, 0.13 dex, and 0.05 dex for Teff, log g, and [Fe/H], respectively. The spectra are selected with the criterion of S/N in g band < 6 in dark nights, and S/N in g band < 15 in bright nights (see,^36^).

Table 1 encompasses a total of $\sim$ 1800 EW systems, however we will focus only on the widely accepted period value for EW (i.e. less than 0.8 day, [e.g.^37^]). The table provides essential details such as system names, types of light curve variability, spectral type, angular separation in arcseconds (between LAMOST and VSX), LAMOST observing date, right ascensions (RA), declinations (Dec), orbital periods per day, effective temperature, log of surface gravity, metallicity, radial velocity, as well as parallax and proper motion with their respective errors. For access to the complete version of the table, please refer to https://zenodo.org/record/8432615.

**Table 1:** Sample data for EW systems.

Name	Type	Sp. type	Ang. dist	Obs. date	RA (deg)	Dec (deg)	Period	Teff	Teff_err	Log(g)	log(g)_err
ZTF J045920.12 + 234803.1	EW	G8	0.063253	2012-12-31	74.83384	23.800865	0.5556214	5461.98	104.47	4.113	0.169
V0788 Mon	EW	K2	0.207901	2012-12-03	111.24707	− 0.4119989	0.43369	5188.02	73.47	4.282	0.12
WISE J045310.9 + 265949	EW	G1	0.120349	2013-02-09	73.295526	26.997157	0.2421656	5814.81	110.16	3.78	0.171
ZTF J043759.37 + 281249.2	EW	G9	0.073829	2013-02-09	69.497398	28.213677	0.237164	4933.35	233.73	3.085	0.382
CSS_J015004.4 + 291126	EW	G3	0.288077	2012-01-13	27.51841	29.190618	0.319273	5683.99	72.34	4.056	0.119
CSS_J014910.9 + 282504	EW	G0	0.542624	2012-01-13	27.295688	28.417814	0.7961344	5898.28	249.9	4.346	0.388
ASAS J081730-0326.1	EW	F5	0.061899	2012-12-22	124.37403	− 3.435926	0.7297574	6317.68	193.1	4.175	0.303
V0372 CVn	EW	K3	0.026328	2013-01-23	205.550218	28.440692	0.5973626	4205.65	84.2	0.736	0.139
V0473 CVn	EW	G5	0.047756	2013-01-23	205.672917	28.318907	0.4341914	4097.67	171.16	0.643	0.27
CSS_J014629.4 + 274739	EW	F0	0.25562	2012-01-13	26.622811	27.794311	0.3464366	6504.94	99.46	4.176	0.164
ZTF J014328.90 + 291916.3	EW	K3	0.144014	2012-01-13	25.870459	29.321189	0.3366088	4732.61	161.89	4.087	0.252
ASAS J044425 + 2237.0	EW	F2	0.145953	2012-12-31	71.102818	22.617151	0.3761574	6795.3	30.91	4.144	0.051
CSS_J013255.0 + 293645	EW	K5	0.315087	2012-01-13	23.229241	29.612683	0.2972792	4532.62	121.85	4.484	0.2
ATO J071.1040 + 23.0066	EW	F0	0.190693	2012-12-31	71.104019	23.00663	0.266596	7513.49	34.96	4.099	0.058
EPIC 202088432	EW	K5	0.477343	2013-02-09	93.693236	26.295478	0.313448	4249.98	105.59	4.625	0.167
CzeV1083	EW	G7	0.046917	2013-02-09	96.890733	27.135594	0.680822	5626.83	203.78	4.217	0.319
KP 105899	EW	K3	0.0108	2013-02-22	133.45578	20.852123	0.371699	4916.13	72.53	4.51	0.119

Name	[Fe/H]	[Fe/H]_err	rv	rv_err	plx	plx_err	pmra	pmdec	pm_err_maj	pm_err_min	
ZTF J045920.12 + 234803.1	0.028	0.1	3.18	9.34	2.0086	0.0181	1.441	− 4.9	0.023	0.016	
V0788 Mon	0.054	0.071	17.7	6.43	2.6063	0.0147	− 18.642	− 13.608	0.014	0.012	
WISE J045310.9 + 265949	0.045	0.102	4.31	10.52	0.9055	0.042	3.954	− 6.057	0.051	0.035	
ZTF J043759.37 + 281249.2	− 0.385	0.223	− 11.02	6.45	0.1953	0.0529	− 0.274	− 1.957	0.069	0.049	
CSS_J015004.4 + 291126	0.018	0.07	− 76.05	14.66	0.7155	0.0219	0.761	− 9.455	0.022	0.024	
CSS_J014910.9 + 282504	− 0.148	0.232	15.75	23.99	0.3451	0.0378	− 0.898	− 2.92	0.044	0.041	
ASAS J081730-0326.1	0.116	0.18	32.8	16.1	0.7104	0.0343	− 6.993	− 1.546	0.039	0.033	
V0372 CVn	− 1.333	0.081	− 163.69	6.03	0.0655	0.0153	− 0.182	− 2.618	0.017	0.01	
V0473 CVn	− 1.14	0.16	− 169.51	4.56	0.0824	0.0168	− 0.165	− 2.586	0.019	0.011	
CSS_J014629.4 + 274739	− 1.303	0.096	− 179.03	15.12	0.1455	0.0325	4.398	− 5.859	0.031	0.029	
ZTF J014328.90 + 291916.3	− 0.458	0.151	− 51.56	15.24	0.9691	0.0684	− 8.168	0.964	0.076	0.049	
ASAS J044425 + 2237.0	0.064	0.029	− 7.85	16.2	2.3463	0.1756	− 5.344	− 8.884	0.206	0.138	
CSS_J013255.0 + 293645	− 0.413	0.118	− 52.74	18.04	1.913	0.0275	5.522	− 30.054	0.029	0.019	
ATO J071.1040 + 23.0066	− 0.168	0.033	83.47	9.88	1.3132	0.0192	11.125	3.771	0.023	0.013	
EPIC 202088432	− 0.114	0.099	− 29.75	2.94	3.6441	0.026	− 7.645	− 30.784	0.024	0.019	
CzeV1083	− 0.057	0.189	22.56	29.17	1.134	0.0224	1.323	0.209	0.026	0.019	
KP 105899	− 0.257	0.07	− 55.42	4.22	4.2176	0.0554	− 5.778	− 3.318	0.058	0.048	

Table 2 presents key statistical parameters for our sample. The mean period is approximately 0.377 with a standard error of 0.003, and the range for this parameter spans from 0.187 to 0.798. The effective temperature ($T_{\text {eff}}$) has a mean value of about 5770 K with a standard error of 20 K, and its range extends from 3860 to 8360 K. Log(g) or Surface gravity’s mean value is approximately 4, with a standard error of 0.017, and the range for this parameter ranges from 0.117 to 4.865. The dataset’s mean metallicity ([Fe/H]) is around − 0.185 with a standard error of 0.009, and its range spans from − 2.273 to 0.566. Finally, the mean radial velocity (RV) is approximately − 4 km/s with a standard error of 1.221, and the range for this parameter is from − 395 to 284 km/s.

**Table 2:** Statistics of the studied parameters within our sample.

No. of sample	Period	$T_{\text {eff}}$	log(g)	[Fe/H]	RV
1781					
Mean $(\bar{x})$	0.377	5773	3.99	− 0.18	− 4.08
Std. error of mean	0.003	21	0.02	0.009	1.22
Median	0.35	5796	4.16	− 0.12	− 3.07
Mode	0.33	5095	4.17	0.08	− 24.58
Std. Deviation (*S*)	0.11	90	0.705	0.37	51.55
Range	0.61	4500	4.75	2.84	680.17
Minimum	0.19	3862	0.12	− 2.27	− 395.55
Maximum	0.79	8362	4.86	0.57	284.62

## Statistical analysis

### Method

Our method for constructing the statistical study of the physical parameters under investigation involves the following steps:

1. Range Calculation: First, we determine the range (R) of the dataset. This is achieved by finding the difference between the maximum and minimum values.
2. Interval Determination: To establish the number of intervals (n) for the frequency distribution,

We adopt Sturges’s rule. This rule is expressed by the equation:

1 $$\begin{aligned} n = 1 + 3.3 \log N \end{aligned}$$

where N represents the total number of data points in the dataset.

3- Interval Length Computation: With the number of intervals (n) determined, we proceed to calculate the interval length (L). This is accomplished using the formula:

2 $$\begin{aligned} L = \frac{R}{n} \end{aligned}$$

where R denotes the range obtained in the first step. By following these steps, we effectively organize the dataset into a meaningful frequency distribution, shedding light on the distribution and variability of the physical parameters. Parameters listed in Table 2 such as the sample size (N), mean $(\bar{x})$, standard deviation $(\sigma )$, minimum, maximum values, and the computed range (R) using our method, are used for this purpose. Details about the method can be found at^38^.

### Frequency distribution

In this subsection, we applied Eqs. (1) and (2) to obtain the number of intervals (*n*) and the interval length (*L*) for each parameter in our sample, resulting in 12 intervals for the frequency distribution. The details of the distributions are presented in the following tables.

#### Period

**Figure 1:**
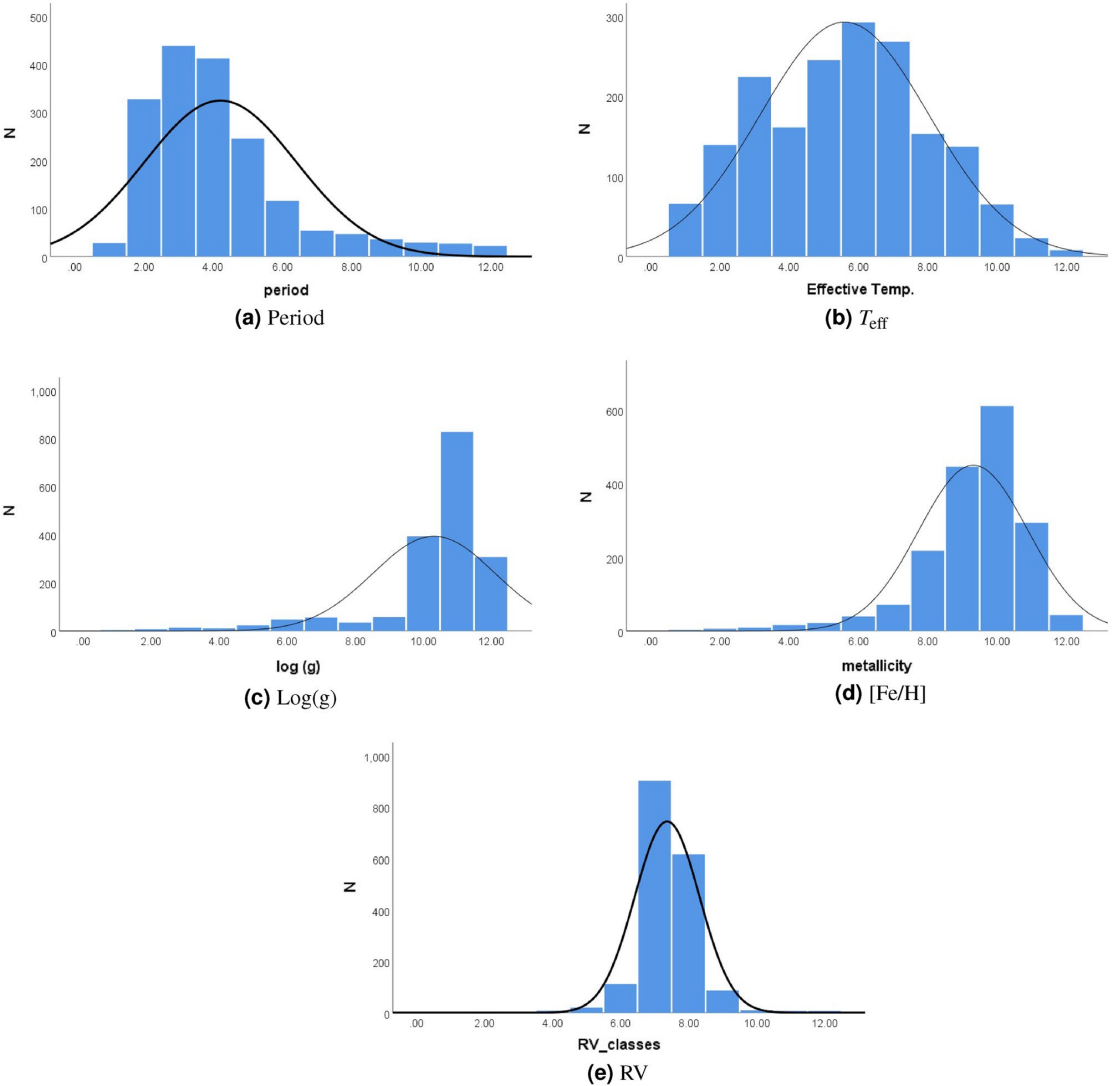
Distribution of EW parameters. The X-axis represents the intervals of the parameter, while Y-axis is the number of Ew systems.

Table 3 illustrates the distribution of the “Period” parameter in our sample. Notably, there are 3 instances with a period value of 0.187, while the majority of data points lie above 0.2. The EW period is highly concentrated within the range of 0.2 to 0.493 (periods less than 0.5), accounting for 1563 data points or approximately 87.8% of the dataset. Furthermore, periods less than 0.6 constitute 1664 data points, representing 93.6% of the dataset. However, in the last four intervals from 0.6 to 0.8, there are only 114 EW cycles, making up approximately 6.4% of the dataset. This finding indicates a significant concentration of the EW orbital period between 0.2 and 0.6, with occurrences above 0.6 being minimal. The corresponding graph (see Fig. 1a) visually illustrates this distribution pattern.

**Table 3:** Preiod frequency distribution.

*n*	*L*	Frequency	Percent (%)
1	0.187–0.238	28	1.6
2	0.238–0.289	327	18.4
3	0.289–0.34	438	24.6
4	0.34–0.391	412	23.1
5	0.391–0.442	245	13.8
6	0.442–0.493	116	6.5
7	0.493–0.544	54	3.0
8	0.544–0.595	47	2.6
9	0.595–0.646	36	2.0
10	0.646–0.697	29	1.6
11	0.697–0.748	27	1.5
12	0.748–0.799	22	1.2
Total		1781	100.0

#### Effective temperature ($T_{\text {eff}}$)

In the $T_{\text {eff}}$ Frequency Distribution” Table (4), we observe significant insights regarding the concentration of temperature degrees ($T_{\text {eff}}$) within the EW sample. Notably, a considerable proportion of $T_{\text {eff}}$ values, totaling 1619 instances, fall within the range of 4236 to 7236, representing approximately 91% of the dataset. On the other hand, the interval from 7236 to 8362 contain a smaller count of only 96 EW systems, accounting for approximately 5.4%. This finding highlights the predominant occurrence of EW temperature degrees between 4236 and 7236, indicating a well-defined concentration in this range. To visually illustrate this distribution pattern, we provide the accompanying figure depicting (Fig. 1b) the frequency distribution of EW temperatures (Table 4).

**Table 4:** $T_{\text {eff}}$ frequency distribution.

*n*	*L*	Frequency	Percent (%)
1	3861–4236	66	3.7
2	4236–4611	139	7.8
3	4611–4986	224	12.6
4	4986–5361	161	9.0
5	5361–5736	245	13.8
6	5736–6111	292	16.4
7	6111–6486	268	15.0
8	6486–6861	153	8.6
9	6861–7236	137	7.7
10	7236–7611	65	3.6
11	7611–7986	23	1.3
12	7986–8362.11	8	0.4
Total		1781	100.0

#### Surface gravity (Log(g))

In the “Log(g) Frequency Distribution” Table 5, it is evident that the highest concentration of log(g) values occurs between 3.68 and 4.87, with a total count of 1524, accounting for 85.6% of the dataset. The remaining 257 instances, comprising 14.4% of the data, are distributed across the other nine intervals. This finding underscores the dominant occurrence of log(g) values between 3.68 and 4.87, as illustrated in Fig. 1c).

**Table 5:** Log(g) frequency distribution.

*n*	*L*	Frequency	Percent (%)
1	0.117–0.513	4	0.2
2	0.513–0.909	8	0.4
3	0.909–1.305	14	0.8
4	1.305–1.701	11	0.6
5	1.701–2.097	23	1.3
6	2.097–2.493	48	2.7
7	2.493–2.889	56	3.1
8	2.889–3.285	35	2.0
9	3.285–3.681	58	3.3
10	3.681–4.077	392	22.0
11	4.077–4.473	826	46.4
12	4.473–4.869	306	17.2
Total		1781	100.0

#### Metallicity ([Fe/H])

The [Fe/H] frequency distribution, Table 6 provides significant insights into the distribution of metallicity values within the EW sample. Notably, the largest distribution of EW systems falls within the range from − 0.14 to 0.097, with a total of 612 systems representing 34.4% of the dataset. This observation indicates that the majority of EW systems in the sample are old stellar population, reflecting their prevalence in the specified metallicity range.

Moreover, a substantial concentration of 1571 EW systems is found within the intervals − 0.614 to 0.334, accounting for 88.2% of the data. The remaining fraction of the dataset, totaling 210 EW systems, is distributed across the other nine metallicity intervals, constituting 11.8% of the sample. Figure 1d displays the [Fe/H] distribution throughout the EW systems of the current sample.

**Table 6:** [Fe/H] frequency distribution.

*n*	*L*	Frequency	Percent (%)
1	− 2.273–(− 2.036)	3	0.2
2	− 2.036–(− 1.799)	6	0.3
3	− 1.799–(− 1.562)	9	0.5
4	− 1.562–(− 1.325)	16	0.9
5	− 1.325–(− 1.088)	22	1.2
6	− 1.088–(− 0.851)	40	2.2
7	− 0.851–(− 0.614)	71	4.0
8	− 0.614–(− 0.377)	218	12.2
9	− 0.377–(− 0.14)	447	25.1
10	− 0.14–0.097	612	34.4
11	0.097–0.334	294	16.5
12	0.334–0.571	43	2.4
Total		1781	100.0

#### Radial velocity (RV)

The “Radial Velocities Frequency Distribution” table reveals distinct patterns in the distribution of radial velocities (RV) for Eclipsing W Ursae Majoris (EW) systems. Over 51% of the EW systems are concentrated in the narrow RV range from − 54 to 3, while approximately 85% are clustered within adjacent categories from − 54 to 60. Moreover, about 92% of the EW systems are distributed in three categories spanning from − 111 to 60, with the remaining categories comprising 8% of the dataset (see, Table 7). Notably, the majority of this 8% is found in the 60 to 117 category. Overall, around 97% of the EW systems fall within four categories ranging from -111 to 117 and beyond.

It is important also to highlight that, RV were observed at different phases and are varying with time. The observations of RV for individual systems (e.g.^39–42^) show that the secondary component exhibits a higher radial velocity than the primary one. This means that in our sample, the higher RV values (e.g. 283 and − 396) can be explained by observing systems’ secondary component. Figure1e visually illustrates this distribution pattern, providing additional clarity on the prevailing trends of radial velocities in our sample.

**Table 7:** RV frequency distribution.

*n*	*L*	Frequency	Percent (%)
1	− 396–(− 339)	3	0.2
2	− 339–(− 282)	4	0.2
3	− 282–(− 225)	3	0.2
4	− 225–(− 168)	8	0.4
5	− 168–(− 111)	20	1.1
6	− 111–(− 54)	112	6.3
7	− 54–3	903	50.7
8	3–60	617	34.6
9	60–117	87	4.9
10	117–174	10	0.6
11	174–231	7	0.4
12	231–288	7	0.4
Total		1781	100.0

#### Spectral types

As mentioned above, Our sample comprises different spectral type including A, F, G and K in this section we are aiming to understand their frequency distribution in addition to the physical properties of each type from the database.

##### Statistical analysis of A spectral type

The binarity of early type stars including A-type by using Lamost is discussed by^43^ and more recently by^44^. They reported that the binary fraction is decreases toward A-type stars. The detection of EWs with A-type stars is not common compared with the later spectral types (i.e. F, G and K)^45,46^. The present sample is originally contains about 21850 A-type stars, only 88 of them are found to be W UMa binaries. Referring to Table 8 for the A spectral type, a notable trend emerges: A significant 44.3% of the observed EWs fall under the category of A7V, amounting to 39 instances. Similarly, A6IV claims 20 occurrences, accounting for 22.7% of the total. These two categories collectively exert a substantial influence, commanding a combined ratio of 67% within this type.

**Table 8:** Frequency distribution of A-spectral types.

A-spectral type	Frequency	Percent (%)
A2V	1	1.1
A3IV	4	4.5
A5V	8	9.1
A6IV	20	22.7
A6V	1	1.1
A7III	3	3.4
A7IV	3	3.4
A7V	39	44.3
A8III	3	3.4
A9V	6	6.8
Total	88	100.0

**Table 9:** Proprieties of A spectral type.

Property	Period	$T_{\text {eff}}$	Log(g)	[Fe/H]	RV
*N*	88	88	88	88	88
Mean	0.375	7400	4.12	− 0.337	− 1.47
Std. error of mean	0.01	44	0.02	0.04	6.01
Std. deviation	0.107	408	0.16	0.38	56
Range	0.537	1861	0.72	2.32	448
Minimum	0.239	6501	3.79	− 1.98	-228
Maximum	0.776	8362	4.51	0.34	220

Presented within this table are the comprehensive descriptive statistics encompassing all parameters specific to type A, comprising a total of 88 EWs instances. Notable observations include the mean period, hovering around 0.375. Comparatively, upon cross-referencing with Table 2, it is apparent that this value remains largely consistent across the spectrum, indicating a uniform mean period across all EWs spectral types. Interestingly, and as listed in Table 9, the mean temperature attains a notably higher value of 7400, which naturally corresponds to the youthful nature of these diminutive stars, characterized by temperatures spanning 7500 to 10,000 K in accordance with the Harvard classification. As for the mean log(g) (4.12), its proximity to the overall sample average of approximately 4 reinforces the coherent tendencies observed throughout the sample. Noteworthy, the mean metallicity registers at − 0.337, contributing an insightful marker of this type’s elemental composition. In the realm of motion, the average radial velocity assumes a value of − 1.5, whereby the negative sign signifies a pronounced blue shift.

##### Statistical analysis of F spectral type

The detection of F-type is believed to be common toward EWs as reported by^47^. They reported that among 90 EWs, 52 systems are classified as F-type. Our results that listed in Table10, indicate that a substantial portion of the EWs population resides within the F0 spectral type, amounting to 239 instances and constituting 40.7% of the dataset. Likewise, F5 captures a significant share of 18.7%, encompassing a total of 128 EWs. In tandem, these two spectral types collectively contribute to an approximate total of 59.4%, highlighting their considerable prevalence among the observed EWs.

**Table 10:** Frequency distribution of F spectral type.

F- spectral type	Frequency	Percent (%)
F0	279	40.7
F2	39	5.7
F3	27	3.9
F4	19	2.8
F5	128	18.7
F6	56	8.2
F7	48	7.0
F8	20	2.9
F9	70	10.2
Total	686	100.0

In the F-type stars, as depicted in Table 11, several noteworthy patterns emerge. The mean period closely approximates the overall mean found in the collective sample encompassing all spectral types. The average temperature, quantified at 6473.6, aligns remarkably well with the Harvard classification’s reasonable range of 6000–7500 K for this type. The mean gravitational acceleration (log(g)) tends to converge towards the overall sample average of 4. Meanwhile, the mean metallicity registers at − 0.191, surpassing the overall sample mean. Notably, the mean radial velocity (RV) stands at − 7.32, underscoring a distinct propensity towards a blue shift for the majority of EWs within this category.

**Table 11:** Proprieties of F spectral type.

parameter	Period	$T_{\text {eff}}$	log(g)	metallicity	RV
N	686	686	686	686	686
Mean	0.371	6474	4.09	− 0.19	− 7.3
Std. error of mean	0.004	17	0.008	0.014	2.213
Std. deviation	0.108	456	0.23	0.37	57.96
Range	0.611	2902	3.45	2.46	645
Minimum	0.187	4897	1.319	− 1.9	− 395
Maximum	0.798	7799	4.76	0.55	249

However, the number of detailed spectroscopic study and RV curves of EWs remains small compared to the known EWs and even compared with our sample, the more recent sample introduced by^14^ exhibiting an average RV of $\sim$ 7.7 km/s with red-shifted. This means that more observations are necessary to better understand the RV nature of EWs.

##### Statistical analysis of G spectral type

In the G spectral type Table 12, it becomes evident that the spectral categories G2, G5, G3, G7, and G8 collectively make up a significant portion, amounting to 74.2% of the dataset and totaling 498 instances of EWs.

**Table 12:** Frequency distribution of G-spectral types.

G-spectral type	Frequency	Percent (%)
G0	32	4.8
G1	35	5.2
G2	75	11.2
G3	124	18.5
G4	25	3.7
G5	127	18.9
G6	30	4.5
G7	96	14.3
G8	76	11.3
G9	51	7.6
Total	671	100.0

Within the spectral type G Table 13 a noteworthy observation emerges: the mean values for period, log(g), and metallicity closely align with the overall sample average. Nonetheless, a distinction arises in terms of radial velocity, deviating from the norm and registering at -1.96. Concurrently, the mean temperature attributed to stars within this spectral type rests at 5408. This value aptly situates itself within the Harvard classification range of 5200–6000, affirming the consistent and accurate spectral classifications for stars within this category.

**Table 13:** Properties of G spectral type.

Parameter	Period	$T_{\text {eff}}$	log(g)	metallicity	RV
N	671	671	671	671	671
Mean	0.384	5408	3.79	− 0.18	− 1.96
Std. error of mean	0.004	19	0.035	0.015	1.87
Std. deviation	0.113	494	0.926	0.397	48
Range	0.609	3112	4.706	2.839	598
Minimum	0.187	3966	0.117	-2.273	-336
Maximum	0.796	7078	4.823	0.566	262

##### Statistical analysis of K spectral type

The spectral type K listed in Table 14 reveals a total count of 336 EWs across various spectral subtypes, excluding K6, K8, and K9. Remarkably, the majority of EWs instances are concentrated within K3, K5, and K7, amassing to a substantial 231 occurrences, constituting a significant 68.75% of the total count.

**Table 14:** Frequency distribution of K-spectral types.

K-spectral type	Frequency	Percent (%)
K0	33	9.8
K1	37	11.0
K2	7	2.1
K3	109	32.4
K4	28	8.3
K5	71	21.1
K7	51	15.2
Total	336	100.0

Within the K-spectral type (see Table 15), we observe that the average values for period and log(g) closely align with the overall sample mean across various spectral types. However, there are notable distinctions in terms of metallicity and radial velocity, registering at -0.144 and -2.4, respectively. The mean temperature within this classification hovers around 4644, effectively situating it within the 3700–5200 range specified by the Harvard classification. This alignment underscores the precise and accurate delineation of spectral classifications within this specific type and catalog.

**Table 15:** Properties of K-spectral type.

Parameter	Period	$T_{\text {eff}}$	Log(g)	[Fe/H]	RV
N	336	336	336	336	336
Mean	0.376	4645	4.185	− 0.144	− 2.416
Mode	0.307	5150	4.661	− 0.397	− 52.74
Std. deviation	0.114	370	0.814	0.274	42
Range	0.568	1985	4.129	2.099	476
Minimum	0.220	3860	0.736	− 1.558	− 190
Maximum	0.788	5850	4.865	0.541	285

### Confidence interval and testing hypothesis

To estimate the confidence interval for the population mean ($\mu$), we utilize the sample mean ($\bar{x}$) and the following equation (see^48^) to determine the confidence intervals for each parameter in the EW systems:

3 $$\begin{aligned} \bar{x} - Z_{(\alpha /2)} \cdot \frac{S}{\sqrt{n}} \le \mu \le \bar{x} + Z_{(\alpha /2)} \cdot \frac{S}{\sqrt{n}} \end{aligned}$$

where *S* represents the standard deviation. The value of $Z_{(\alpha /2)}$ is determined based on the confidence level (or significance level), and in this study, the confidence level is set at 95%, resulting in $Z_{(\alpha /2)} = 1.96$.

After calculating the confidence intervals for each parameter, the next step involves hypothesis testing for these parameters. The conditions for these tests are as follows:

1. The variable should follow a normal distribution, although this condition can be disregarded if the sample size is large ($N > 30$).
2. The sample should be random, and the values of its individuals should be independent of each other. (Both of these conditions are met in this study).

The probability value (p-value) which serves as a crucial tool for statistically assessing hypotheses becomes discernible when the p-value exceeds 0.05 (5%), a threshold commonly known as the significance level. By applying Eq. (3), we calculate the 95% confidence interval for the mean of the studied parameters ($\mu _i$) for EWs as follows:

4 $$\begin{aligned} a \le \mu _i \le b \end{aligned}$$

where a and b in Eq. (4) represent the interval’s limits for the parameter i.

To determine whether the limit of the inequality obtained in relation (4) is acceptable or not, we performed hypothesis testing as follows:

$$H_0: \mu _i = a$$ versus $$H_1: \mu _i \ne a$$

$$H_0: \mu _i = b$$ versus $$H_1: \mu _i \ne b$$

In these statistical hypotheses, $H_\text {0}$ represents the null hypothesis, while $H_\text {1}$ represents the alternative hypothesis.

Table 16 summarizes the results of our testing hypotheses of the studied parameters.

**Table 16:** The 95% confidence interval and the corresponding P-value of the EWs parameters.

Parameter	95% confidence interval	P-value
Period	0.372 to 0.382	0.053
$T_{\text {eff}}$	5730 to 5820	0.051
Log(g)	3.97 to 4.03	0.062
[Fe/H]	− 0.20 to − 0.17	0.054
RV	− 6.47 to − 1.7	0.051

Upon analyzing the P-value, it is evident that the values are greater than 0.05 for all parameters. As a result, we accept the null hypothesis, indicating that the mean values for EWs systems falls within the limits defined by the inequality (4).

**Table 17:** Correlation between the studied sample of EW’s parameters.

Correlations	Period	$T_{\text {eff}}$	$\log (g)$	[Fe/H]	RV
Period	1	− 0.024	− 0.041	0.015	− 0.039
$T_{\text {eff}}$	− 0.024	1	0.249	0.12	− 0.053
$\log (g)$	− 0.041	0.249	1	0.394	− 0.104
[*Fe*/*H*]	0.015	0.12	0.394	1	− 0.012
RV	− 0.039	− 0.053	− 0.104	− 0.012	1

Periods are taken from VSX catalog.

**Table 18:** Correlation between the studied sample of EW’s parameters.

Correlations	Period	$T_{\text {eff}}$	$\log (g)$	[Fe/H]	RV
Period	1	0.74	− 0.249	0.170	0.008
$T_{\text {eff}}$	0.74	1	− 0.231	-0.047	0.11
$\log (g)$	− 0.249	− 0.231	1	0.083	0.006
[*Fe*/*H*]	0.170	− 0.047	0.083	1	0.019
RV	0.008	0.110	0.006	0.019	1

Periods are taken from ZTF catalog.

### Statistical relation between EW’s parameters

**Figure 2:**
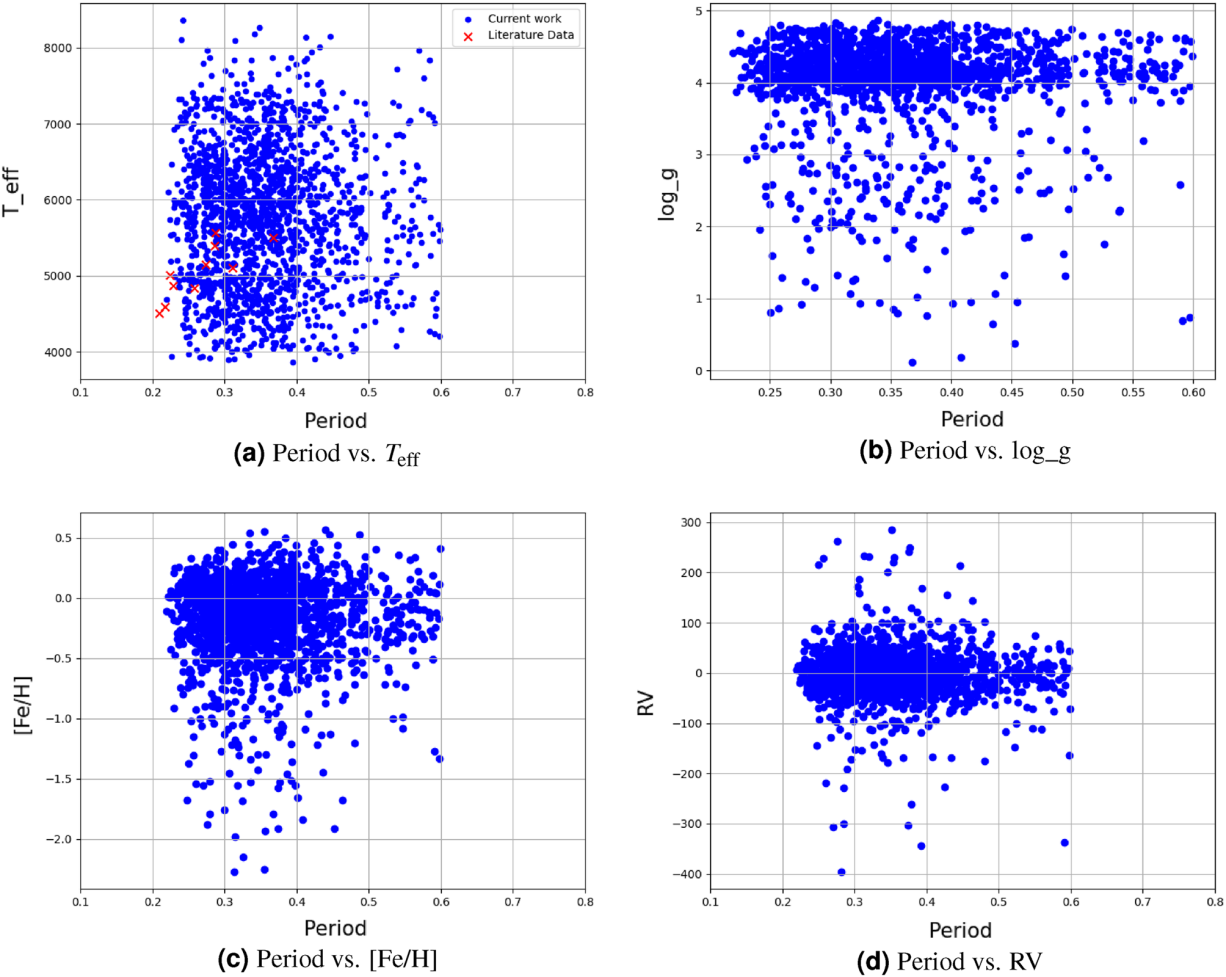
Period from VSX catalog vs. various parameters of EW systems.

**Figure 3:**
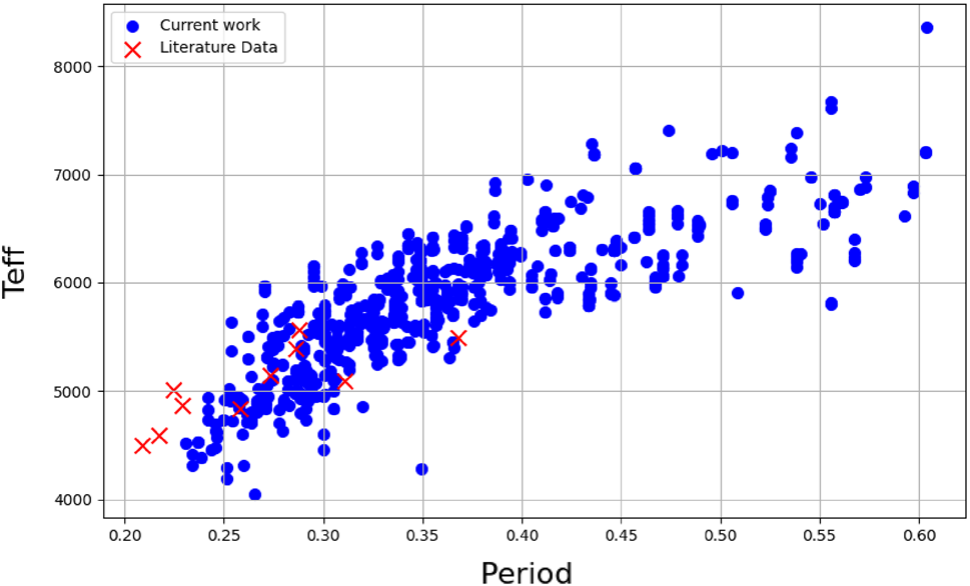
Period from ZTF catalog vs. Teff of the EW systems.

In this section, we investigate the correlation between periods obtained from the VSX and ZTF catalogs, a crucial parameter for tracing the evolutionary status of EW systems, along with other parameters such as $T_{\text {eff}}$, Log(g), [Fe/H], and RV. Considering the period’s range in our sample (see Section “Period”), limited to 0.2 to 0.6 days, we explore the relationship with these parameters. Initiating with VSX catalog periods, Fig. 2 and Table 17 indicate no significant correlation with other parameters, particularly the period-$T_{\text {eff}}$ relation, deviating from the well-established relation introduced by^49^. To address this, we turn to the ZTF catalog, the sample’s cross-matching resulted in a list of 345 confirmed EWs, of which 315 have periods < 0.6 days. Table 18 reveals a strong correlation between period and $T_{\text {eff}}$, as illustrated in Fig. 3 with an upward trend. Comparing our dataset with literature values (refer to Table 19), depicted in Figs. 2 and 3, indicates alignment with previously estimated values.”

**Table 19:** Sample of studied EW systems with Period, $T_{\text {eff}}$, and the corresponding Reference.

Period	$T_{\text {eff}}$	Reference
0.20908	4500	^4^
0.2864	5392	^5^
0.228882	4872	^7^
0.28781	5567	^7^
0.21751	4589	^50^
0.2581	4838	^51^
0.3107068	5100	^52^
0.367992	5500	^52^
0.273553	5150	^53^
0.224734	5010	^54^

## Discussion and conclusion

In this work we have presented a catalogue of $\sim$ 1800 EWs based on LAMOST, VSX and Gaia parameters. A details statistical analysis including: parameters distribution, confidence intervals and testing hypotheses to enable understanding the physical properties of such important eclipsing binary class.

In our catalog, we focused on several key parameters, including Period, Effective Temperature, Log(g), [Fe/H], and Radial Velocity, as well as the spectral type of the systems. Our study revealed that for EW systems, the mean period is 0.377 days and with 95% confidence, the majority falling within the range of 0.372 to 0.382 days. The mean effective temperature is approximately 5773 K, with most EW systems falling within the range of 5730 to 5820 K. The average metallicity is estimated to be − 0.185, and the majority of systems fall within the range of − 0.202 to − 0.168. The mean log of surface gravity for EW systems is approximately 4, with most samples ranging from 3.97 to 4.03. The average radial velocity for EW systems is − 4.085 km/s, within the range of − 6.47 to − 1.7 km/s.

Our study also confirms that the majority of EW systems are Late-type stars, primarily classified as F spectral type, followed by G and K. Among the sample, 88 systems are classified as A spectral type, with a mean surface temperature of 7400 K (i.e. stars with radiative envelopes). These findings could suggest that A-spectral type systems may not be classified as typical EW systems and they need a further investigations for better classifications.

To the best of our knowledge, this study represents the first instance of introducing confidence interval limits at a 95% confidence level for the atmospheric parameters of the EWs. Additionally, we conducted hypothesis testing based on these limits. However, prior research on general statistical properties of EWs has been undertaken by others, including studies by^20^ and^55^. The authors in^20^ focused on identifying peaks in the distribution of studied parameters and determined that the period, $T_{\text {eff}}$, log(g), RV, and [Fe/H] exhibited peaks around 0.29 days, 5700 K, 4.16, − 20 km/s, and − 1.5, respectively. While our findings align with theirs for $T_{\text {eff}}$, log(g), and [Fe/H], there are deviations in the observed periods and RV. Our study possesses the advantage of conducting a spectral type distribution analysis for EWs, leading to the conclusion that F-spectral types dominate among the various late-type systems.

On a different note^55^, collected data from approximately 700 previously analyzed systems to conduct a statistical investigation, focusing on parameters such as period, $T_{\text {eff}}$, mass ratio, and the system’s age. Their findings indicated that 50% of EWs have periods between 0.28 and 0.43 days, with a mean value of 0.35. Our results are comparable, as we observed that around 50% of our sample falls within periods ranging from 0.289 to 0.391, with a mean value of 0.34. They reported a mean $T_{\text {eff}}$ of approximately 5760 K, which closely matches our results (5770 K).

The correlation between the orbital period and the atmospheric parameters from the VSX and ZTF catalogs has been assessed. A strong agreement is observed, except for the period-Teff relation. Our findings indicate that ZTF periods align well with previously published relations, showing a correlation coefficient of 0.74. In contrast, a weak correlation is observed in the periods-Teff relation from the VSX catalog. This suggests a need for revising the VSX periods, as they may not be accurately recorded for the studied sample of EWs. In conclusion, our study enriches our understanding of Eclipsing W UMa systems by introducing confidence interval limits with hypothesis testing and focusing on spectral type distribution. This unique approach sets our work apart, providing a more comprehensive insight into this crucial class of eclipsing binaries. These findings not only advance our knowledge of EW systems but also open avenues for further investigations into their diverse characteristics, classifications, and evolutionary status.

## Data Availability

The data underlining this work is available at https://zenodo.org/record/8432615.
